# Evolution of Dentistry

**Published:** 1891-03

**Authors:** J. A. Robinson


### Evolution of Dentistry.

Embracing Remarks made at the "New York Banquet of Veteran Dentists," January 31, 1891.

BY J. A. ROBINSON, D.D.S.

We are now fast approaching a line of a profession, and profession means brotherhood. As dentistry began generally with personal friendships coupled with some mechanical skill and a few tools, and the desire to assist those who were afflicted by the loss of their teeth through accident, neglect or hereditary proclivities; and largely as a matter of sympathy for the suffering, the attempt was made to aid to a more comfortable life; and dentistry was born. In the beginning each individual was isolated and worked alone by himself and for himself, but as the demand grew the thought grew, friendship grew, and concert of action, and education, and science and higher laws presented themselves, that commanded the attention of those most devoted to their calling, and by a law of nature strictly in accordance with evolution; as all the improvements in civilization have come since the primitive man saw a God in every object in nature that he could not comprehend, and so by a law of progress, until to-day we have reached a time when we see one overruling power holding all worlds, so to speak, in the hollow of his hand. And so we see all this individual labor of the past fifty years in our own calling corresponding and forming a common brotherhood, and striving to raise the members to a oneness that shall correspond to our ideas as a Creator, that we may go forward to higher aims and loftier achievements. The three subjects upon which we are not united and on which there is the greatest diversity of opinion, at this time, are artificial crowns, bridgework and pyorrhoæ alveolaris. The capping of exposed pulps is assured if done with care by good operators; it is no longer empyrical or guess work. These three subjects have been evolved out of the growth of dentistry as a scientific study and pursuit. The fitting of the band is the most important thing in crowns and bridge work, after removing the enamel at the gum and leveling the root so the band will slide as accurately as the section of the telescope. To get the exact measure for the band take a fine wire thread, three inches long, and loop it in the middle by twisting the two ends together 'till it is almost small enough to go over the end of the root; now take a watchmaker's sliding pin vice and take hold of the twisted end of the wire; slip the loop over the root and pull the wire gently against the posterior side and twist the loop 'till it is tight all around; this can be told, as when it is all tight the wire will kink; take it off and cut the wire opposite the twist and straighten it, and you have the exact measure of the root. The advantage of this way over the pliars generally used, is that the left hand is free to hold the wire in position, and the right hand has constant hold of the wire, and it is always away from the lips, and the back teeth are measured as easily as the front teeth and can be measured across the mouth on one as well as the other.

It will often be a cause of disappointment to any dentist who undertakes to do crown or bridge work with porcelain facings, without the protection of gold to prevent the breaking; to do this, after you have fitted the teeth a little short of articulation, square the ends or cutting edges, back them with thin platinum, bend the pins and do not let the platinum come over the end of the tooth; now take a piece of gold plate, 20 in thickness and large enough to cover the end of the tooth, and put it up to the end of the backing, wax it in place, bed it in plaster and solder as is usually done in backing a tooth; this will make a beautiful finish and serve as protection when you solder for the final finish.

If you have a broken tooth and do not wish to remove the bridge, select a tooth that will not quite reach to the old backing; now take a strip of very thin platinum and wide enough for a baking and leave the ends long crosswise and flow gold over one side and back, and solder to the pins; now square the old backing that is on the bridge, put the tooth in place and bring the long ends together round the old backing, now remove the tooth and fill the slot with yellow ochre to keep the solder out, and solder the bands together; this will leave a pocket to receive the old backing that is on the bridge and fasten it in place with amalgam. The object of flowing gold over the platinum is so the amalgam will unite with it, as it will not unite with platinum. When a root is very short take a fine hairpin and bend it in form of a button hook, barb the long end and cement it in the root before cementing on the crown, or if the root is sufficiently strong make a staple and anchor into the canal, and your crown will never come off. A hairpin is best because it is covered with a varnish that prevents rusting with the cement.

If a crown is to be attached where the pulp is not exposed, cut two very fine horizontal grooves around the root to hold the cement, as it always leaves the tooth before it will come out of the band. What are known as Richmond crowns are par excellence for artificial substitutes, removable bridge work is next; and the permanent bridge is safe practice where we have an anchor at both ends of the bridge.

I have experimented with the newly patented plan of Dr. N. I. Goodwin, of Hartford, Conn. I have experimented with it some years ago with every prospect of success, but they were entire failures.

In pyorrhoæ I know of nothing that will compare with carbolized potash known as Robinson's Remedy to effect a permanent cure, and it is the best thing I have ever used as an obtundent for sensitive dentine. I introduce two letters, from patients* who had the worst cases I have ever seen, that will speak for themselves. After applying the remedy according to directions, I pack the gums with carbonate of lime which is an antacid, absorbent and astringent, and press the gums close around the teeth to keep out all secretions, and if you have been thorough enough with your chisel to remove all calcarious deposit and produce a new wound at the alveolus, it will heal by first intention, if the secretions and air are kept out, for all life and cure comes from within. I have cured a number of molars where the absorptions

* The letters here referred to give evidence that the treatment of the teeth of the writers was entirely successful, in restoring the teeth to firmness and usefulness.—Ed.

had been so great that I could pass a small instrument between the bifercated roots, but I could not restore the gums. The teeth remained quite firm without any artificial support. In treating such cases I tie a piece of floss silk to the cotton rope, saturated with the remedy, to fish it out when the medicine has done its work. I also give a box of carbonate of lime for the patient to pack the teeth and gums every night before going to bed, as the gums are more liable to infection while in repose than during the day ; also insist on thorough brushing at night before packing the gums, as bacteria are more liable to find a lodgment dur ing sleep. Wounds of the soft tissue must be kept air tight to heal by first intention, as the process of granulation is very slow if disturbed by mastication. We must follow nature's process of cure, if we expect to achive success. When the earth was undergoing gestation, in the fullness of time, trees and plants came forth to fit it for the abode of man, then man was born. The primitive man has been making mistakes and blundering for centuries and his modes of living have brought destruction to his teeth, and at last man resorted to mechanical contrivances for self-preservation and relief. As the necessities grew upon the race, new methods of investigation rose with the occasions, chemical affinities were discovered and when the sympathetic character became coupled with the opportunity a series of efforts grew into the occasion, to give life and permanence to a new profession; then dentistry as a profession was born. Those who have been working for half a century or more can recollect when the germs of what is now a noble profession sprang into being. When we began to work, our failures and our difficulties suggested new methods and modes of practice; then by transformation and transmutation, and genius, and diligence, and industry we advanced step by step, gaining only a little the first twenty-five years. Then came the rubber dam, the dental engine, the cements, the improved disks, the mallet, and painless extraction of the teeth and cohesive foil seemed almost born in a single year. Colleges were endowed and we were enrolled among the learned professions. Civilization and the arts, science and morals are twin brothers of the same great family. " All are but parts of one stupendous whole, whose body nature is and God the soul." It is the same with mechanics and professions. Man is never quite satisfied when his physical wants are all supplied he is striving for something more; we want something higher and better, and all his wants are born out of a previous condition. It is like the birth of water. When hydrogen and oxygen are brought together then water is born, and the new birth brings plants, leaves and flowers to correspond to the surroundings that it took ages of the earth's rotation and gestation to produce. The sliding scale may consume ages but the leaves, the plants and flowers are a new creation. So it required a higher culture and civilization to bring forward our beautiful profession. The recent discussion about contour and face fillings is easily disposed of; when the axis of the tooth corresponds with the axis of the occluding tooth or teeth it will never tip forward or backward, and it is the same with artificial dentures.

And now at the close of this Nineteenth Century we behold a profession born within the century, and within the memory of these few witnesses who have left their homes to be guests of their professional brothers of the State of New York; we urge you as the fathers of the profession to hand it down to the present generation with all the growth and improvements to the younger members in true professional character, to grow higher and broader to the end of time.

---

CHLOROFORM IN CONVULSIONS.—Inhalations of chloroform are invaluable in arresting convulsions in children of two years or under. Give them a whiff of it while in the convulsion. The paroxysm having been broken, the following should be given to prevent recurrence:

R. Sodii. bromidi.........................j drachm.

Chloral hydrat.........................½ ounce.

Aq. menthæ pip.......................

Syr. tolutani a a.......................jv fluid drachms.

Misce. Signa. One-half teaspoonful every half hour until child is quieted down.—*Times and Register*.

